# Critical Roles of p53 in Epithelial-Mesenchymal Transition and Metastasis of Hepatocellular Carcinoma Cells

**DOI:** 10.1371/journal.pone.0072846

**Published:** 2013-09-02

**Authors:** Zheng Wang, Yuhui Jiang, Dongxian Guan, Jingjing Li, Hongkun Yin, Yi Pan, Dong Xie, Yan Chen

**Affiliations:** Key Laboratory of Nutrition and Metabolism, Institute for Nutritional Sciences, Shanghai Institutes for Biological Sciences, Chinese Academy of Sciences, University of Chinese Academy of Sciences, Shanghai, China; Institute of Hepatology, Foundation for Liver Research, United Kingdom

## Abstract

Hepatocellular carcinoma (HCC) is one of the most malignant tumors and the biggest obstacle in curing HCC is its high metastasis potential. Alteration of p53 is the most frequent genetic change found in HCC. Although the biological function of p53 in tumor initiation and progression has been well characterized, whether or not p53 is implicated in metastasis of HCC is largely unknown. In this study, we analyzed the potential functions of p53 in epithelial-mesenchymal transition (EMT) and metastasis of HCC cells. Both insulin- and TGF-β1-induced changes of critical EMT markers were greatly enhanced by p53 knockdown in HCC cells. The insulin- and TGF-β1-stimulated migration of HCC cells were enhanced by p53 knockdown. Furthermore, *in vivo* metastasis of HCC cells using different mouse models was robustly enhanced by p53 knockdown. In addition, we found that p53 regulation on EMT and metastasis involves β-catenin signaling. The nuclear accumulation and transcriptional activity of β-catenin was modulated by p53. The enhanced EMT phenotype, cell migration and tumor metastasis of HCC cells by p53 knockdown were abrogated by inhibiting β-catenin signal pathway. In conclusion, this study reveals that p53 plays a pivotal role in EMT and metastasis of HCC cells via its regulation on β-catenin signaling.

## Introduction

Hepatocellular carcinoma (HCC) is currently the sixth most common cancer and the third leading cause of cancer-related death in the globe [1]. The major risk factors of HCC are chronic hepatitis virus infection, aflatoxin B1 exposure and alcohol use with a stark difference by geographic localization. Due to the nature of high vasculature of the liver, HCC is prone to both intrahepatic and extrahepatic metastases that are the main cause of treatment failure. Studies on the molecular pathogenesis of HCC have revealed that a large array of genetic changes occurs during the development of HCC, including genes implicated in signaling pathways such as those mediated by p53, Ras/ERK, PI3K/AKT, and *wnt*/β-catenin [2]. However, although the molecular pathogenesis of HCC has been greatly elucidated in recent years, most of these studies are limited to the functions of the underlying signaling pathways during the initiation and progression of HCC. The functional roles of these signaling pathways in tumor metastasis are largely elusive.

Accumulating studies have demonstrated an important link between epithelial-mesenchymal transition (EMT) and the invasion and metastasis of cancer cells [3]. EMT is a biologic process in which polarized epithelial cells undergo multiple internal biological changes to transit into a mesenchymal phenotype which is featured with fibroblastoid-like shape, highly mobile and invasive capacity [4]. It has been recognized that EMT plays a pivotal role in the progression and metastasis of HCC [5]. Among all the known factors involved in EMT, TGF-β- and *wnt*/β-catenin-mediated signaling pathways have taken a center stage [5]. Binding of TGF-β family ligands to their receptors leads to subsequent phosphorylation of Smad2/3, which are then translocated into the nuclei after forming a complex with Smad4 to regulate the expression of transcription factors implicated in initiating EMT program, including Snail, Twist, Zeb1, and Slug [3], [6]. These transcriptional factors can bind to the E-box of E-cadherin promoter region to repress the expression of E-cadherin, a major component of epithelial adherens junctions [7]–[11]. On the other hand, β-catenin, a major player in *wnt* signaling, is a multifunctional protein that plays an essential role in cell adhesions [12]. Cytoplasmic β-catenin forms a complex with E-cadherin and is involved in maintaining cell-to-cell contact of epithelial cells [13]. Nuclear accumulation of β-catenin driven by stimuli such as *wnt* enhances β-catenin binding with transcription factor TCF/LEF and such complex subsequently regulates transcription of specific genes involved in EMT including Snail and vimentin [14], [15]. In addition, it has been demonstrated that β-catenin can collaborate with PI3K/AKT pathway to enhance EMT in HepG2 cells [16]. The crosstalk between PI3K/AKT andβ-catenin pathways is mainly achieved by inactivation of GSK3 upon PI3K/AKT stimulation, thus blocking degradation of β-catenin and enhancing the signaling cascade downstream of β-catenin [16], [17].

The gene of p53 is a well characterized tumor suppressor gene and is either lost or mutated in about half of all human cancers [18]. In HCC, p53 is the most frequently mutated gene, resulting in either loss of function or gain of new function [19]–[22]. Previous studies on p53 are mainly focused on its role in the regulation of cell cycle, apoptosis and genomic stability [23]. It is well established that loss or inhibition of p53 function prevents cellular apoptosis and contributing to HCC development [24]. However, whether p53 plays a functional role in EMT and tumor metastasis has not been elaborated. In this study, we analyzed the potential function of p53 in the regulation of EMT and metastasis of liver cancer cells. Our studies reveal that p53 plays a pivotal role in orchestrating the signaling pathways of PI3K/AKT, TGF-β and β-catenin to control EMT and metastasis of HCC cells.

## Materials and Methods

### Cell culture and chemicals

The human portal vein tumor thrombus of hepatocellular carcinoma cell line PVTT-1 stably expressing a luciferase-coding sequence was described previously [25]. PVTT-1, HepG2 and Hep3B cells were cultured in Dulbecco's Modified Eagles Medium (GIBCO, Carlsbad, CA, USA), supplemented with 10% fetal bovine serum. Insulin and TGF-β1 were from Sigma-Aldrich (St. Louis, MO, USA). XAV939 was from Tocris Bioscience (Bristol, United Kingdom).

### Plasmids, cell transfection, lentivirus infection, cell sorting, and immunofluorescence study

The full length cDNA of human β-catenin-interacting protein (ICAT) was cloned by RT-PCR using cDNA isolated from human HCT116 cells and subcloned into a pRc/CMV-based vector in which a Flag epitope tag was added at the N-terminus. The construction of human p53 plasmid was described previously [26]. Lentivirus with p53 shRNA was purchased from GenePharma (Shanghai, China). The target sequences for p53 was 5′-GAAACCACTGGATGGAGAATA-3′ which was selected from four different target sequences as previously described [26]. Both of the shRNA sequence for p53 and ICAT coding region were inserted into the PGPU6/GFP/Neo vector (GenePharma). Transfection in Hep3B cells was performed using polyethylenimine (PEI) method [27]. Transfection of the lentivirus into PVTT-1 and HepG2 cells was conducted under the instruction of GenePharma Recombinant Lentivirus Operation Manual (GenePharma). At 72 h after lentivirus transfection, GFP-positive PVTT-1 cells were sorted by flow cytometry (BD Biosciences, Franklin Lakes, NJ, USA) to select for cells with stable expression of p53 siRNA or ICAT. The protocol for immunofluorescence staining has been described previously [27].

### RNA extraction and real-time RT-PCR analysis

Methods of RNA extraction and real-time RT-PCR analysis have been previously described [26]. The primers used in PCR are listed as follows: 5′-CAGCACATGACGGAGGTTGT-3′ and 5′-CCAGACCATCGCTATCTGAGC-3′ for human p53, 5′-CCTATGCAGGGGTGGTCAAC-3′ and 5′-CGACCTGGAAAACGCCATCA-3′ for human ICAT, 5′-AAGCATTTCAACGCCTCCAAA -3′ and 5′-GGATCTCTGGTTGTGGTATGACA-3′ for human Snail, 5′- GTCCGCAGTCTTACGAGGAG -3′ and 5′- GCTTGAGGGTCTGAATCTTGCT -3′ for human Twist, 5′- GATGATGAATGCGAGTCAGATGC -3′ and 5′- ACAGCAGTGTCTTGTTGTTGT -3′ for human Zeb1, 5′-GATCATTGCTCCTCCTGAGC-3′ and 5′-ACTCCTGCTTGCTGATCCAC-3′ for human β-actin.

### Immunoblotting and antibodies

The protocol for immunoblotting has been described previously [27]. The band intensity was quantified by arithmetic analysis using the software Quantity One 4.5.0. The antibodies used were as follows: E-cadherin and β-catenin from BD Transduction Laboratories (San Jose, CA, USA); ZO-1, Zeb1, Snail, p-AKT, T-AKT, and phospho-Smad2/3 from Cell Signaling Technology (Danvers, MA, USA); vimentin from Bioworld Technology Inc. (St. Louis, MN, USA); total Smad2/3, p53 and tubulin from Santa Cruz Biotechnology (Santa Cruz, CA, USA); TBP from Abcam (Cambridge Science Park, Cambridge, UK).

### Luciferase assay

LEF-1 luciferase reporter has been described previously [28]. Hep3B cells were seeded in 24-well plates before the day of transfection. Cells were transfected with 30 ng of LEF1 reporter plasmid, 10 ng of LEF1 expression plasmid, 0.5 μg N-Flag tagged p53 and/or 0.5 μg N-Flag-tagged ICAT per well, together with 50 ng of renilla luciferase vector as an internal control for transfection efficiency. The dual luciferase assay was described previously [29].

### Wound-healing and transwell assay

The protocols for wound-healing assay and transwell assay were described previously [30]. For wound-healing assay, the width of the wound at 0d h and 48 h were analyzed by Image-Pro Plus5.0 software (Media Cybernetics, Bethesda, MD, USA). Cell migration parameters were calculated in pixels as wound closure of the width at 48 h minus that at 0 h. For transwell assay, images of eight fields were obtained using an OLYMPUS upright microscope. The migrated cells were quantified by image-pro plus 5.0 and the area of migrated cells per field was quantified and calculated in pixels to evaluate the migration ability.

### *In vivo* systemic metastasis assay

All mice used in our research were male BALB/c nude mice (purchased from SLAC laboratory animal company, Shanghai, China) which were kept and used under the guidelines of the Institutional Animal Care and Use Committee of the Institute for Nutritional Sciences, Chinese Academy of Sciences, with free access to standard mouse chow and tap water housing in IVC under SPF environment. All of the experimental procedures were carried out in accordance with the CAS ethics commission with an approval number 2010-AN-8. The mice were divide into 4 groups randomly with each group have 8 mice. FACS-selected and lentivirus-infected PVTT-1 cells with p53 knockdown and/or ICAT overexpression were injected into the left ventricle of each six-week-old mouse (2×10^5^ cells/mouse in 100 μl PBS). Luciferase expression was determined by using intraperitoneal injection of 150 mg/kg D-luciferin (Caliper, Hopkinton, MA, USA), followed by analysis in an *in vivo* imaging system (Xenogen, Hopkinton, MA, USA). The detailed procedure had been described previously [25]. Successful injection was confirmed within 15 minutes after injection by luciferase expression via *in vivo* imaging. Tumor metastasis events were determined in 4 weeks after the injection. The images were captured by Living Image 2.60 and analyzed by Igor Pro 4.09A software (Xenogen, Hopkinton, MA, USA). Hematoxylin-eosin (H&E) staining and immunohistochemistry of the liver tumor sections were performed as previously described [26].

### Orthotopic liver metastasis assay

Five-week-old male BALB/c nude mice were divided into 4 groups randomly with each group have 5 mice. Lentivirus-infected and FACS-selected PVTT-1 cells with p53 knockdown and/or ICAT overexpression were mixed with 20 μl matrigel matrix (BD Biosciences) and slowly injected into the left hepatic lobe of the mice (4×10^5^ cells/mouse in 20 μl PBS) after midline laparotomy. Successful injection was confirmed 2 days after injection by luciferase expression via *in vivo* imaging. Luciferase determination method has been described in systemic metastasis assay. Intrahepatic metastatic foci in hepatic lobes other than the injected lobe were determined at 6 weeks after the injection after sacrifice of the animals.

### Statistical analysis

*Student*'s t test was performed for all the data.

## Results

### Knockdown of p53 enhances TGF-β1- and insulin-stimulated EMT in liver cancer cells

**Figure 1:**
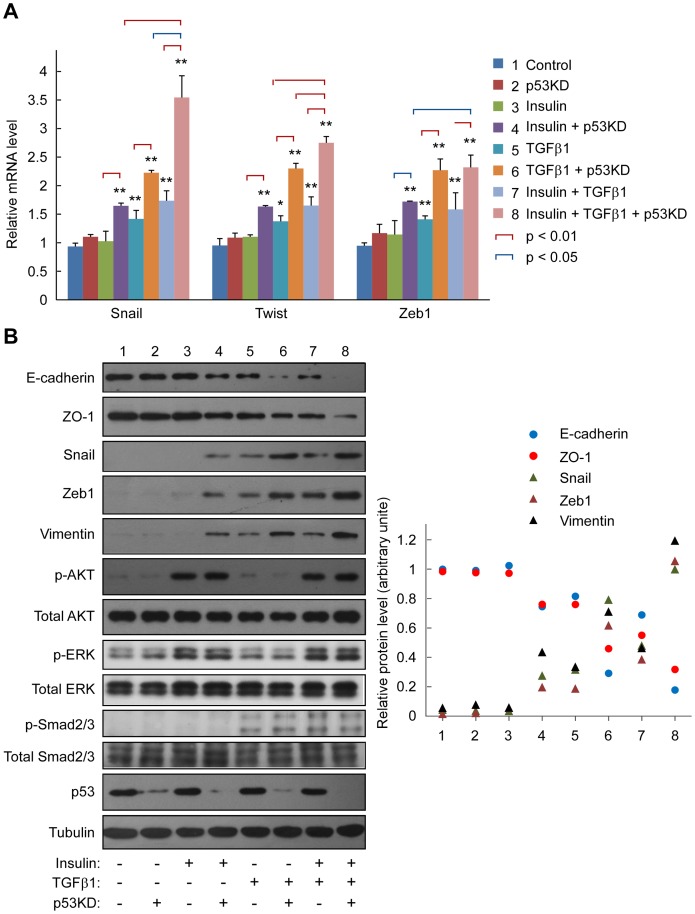
TGF-β1- and insulin-induced EMT is enhanced by p53 knockdown. PVTT-1 cells with or without p53 knockdown (p53KD) were treated with insulin (10 μg/ml) and/or TGF-β1 (5 ng/ml) for 24 h before analysis using realtime RT-PCR to determine the mRNA levels of Snail, Twist and Zeb1 (**A**). The data are calculated from triplicate experiments and shown as mean ± SD. The cells treated with or without insulin and/or TGF-β1 for 48 h were also used in immunoblotting with the antibodies as indicated (**B**). Relative band intensity of E-cadherin, ZO-1, Snail, Zeb1, and vimentin from three independent experiments was calculated and shown on the right.

As previous studies have demonstrated that both TGF-β and PI3K/AKT pathways are critical for EMT in cancer cells including those from HCC [3], [5], [16], we firstly analyzed whether p53 has a functional role on TGF-β- and PI3K/AKT-induced EMT in PVTT-1 cell. PVTT-1 is a cell line obtained from a 60-year-old male HCC patient accompanied by portal vein tumor thrombus (PVTT) and it has an aggressive phenotype in terms of cell growth, survival, migration and metastasis [25], [31]. PVTT-1 cells contained a wild type p53 as confirmed by sequencing. TGF-β signaling was stimulated by treatment of the cells with TGF-β1, while PI3K/AKT and ERK were activated by insulin administration (Figure 1A). The expression of p53 was silenced by infection of the cells with a p53-specific shRNA lentivirus as previously reported by us [26]. We found that the p53-sepcific shRNA lentivirus could reduce p53 mRNA level to ∼27% as compared with the cells transfected with the control lentivirus (Figure S1 in File S1). The EMT status of the cells was analyzed by measurement of the mRNA levels of three representative EMT markers including Snail, Twist and Zeb1 (Figure 1A). When PVTT-1 cells were treated with insulin or TGF-β1 alone, none of them could significantly induce the expression of these markers. Combined treatment of the cells with insulin and TGF-β1 could induce expression of Snail and Twist, indicating the PI3K/AKT and TGF-β signaling pathways have a synergistic effect to promote EMT in these cells. Interestingly, while p53 knockdown alone had no effect on the expression of these three EMT markers (Figure 1A), knockdown of p53 could robustly enhance the insulin- and TGF-β1-induced expression of these EMT markers.

We next analyzed the protein levels of Snail and E-cadherin which is considered as one of the most important factors involved in EMT [32]. As expected, insulin was able to stimulate AKT phosphorylation and TGF-β1 was capable of increasing Smad2/3 phosphorylation (Figure 1B), confirming that both insulin and TGF-β1 were able to initiate their downstream signaling events in PVTT-1 cells. Meanwhile, p53 knockdown alone could not alter the protein levels of a series of EMT markers including E-cadherin, ZO-1, Snail, Zeb1 and vimentin (Figure 1B). Interestingly, p53 knockdown could apparently synergize with insulin and TGF-β1 to reduce the expression of epithelial markers E-cadherin and ZO-1. Furthermore, E-cadherin and ZO-1 reached the lowest expression level when the cells were treated with insulin and TGF-β1 combined with p53 knockdown. Consistently, p53 knockdown could apparently enhance the expression of mesenchymal markers Snail, Zeb1 and vimentin stimulated by insulin and TGF-β1. In addition, in another liver cancer cell line HepG2 derived from hepatoblastoma, p53 knockdown together with insulin and TGF-β1 treatment could cause maximal reduction of E-cadherin and ZO-1 expression and stimulation of Snail, Zeb1 and vimentin expression (Figure S2 in File S1). Collectively, these findings indicate that p53 knockdown can bring about maximal EMT phenotype that is initiated by activation of TGF-β and PI3K/AKT signaling cascades in the liver cancer cells.

### Nuclear accumulation of β-catenin and its transcriptional activity is negatively regulated by p53

**Figure 2:**
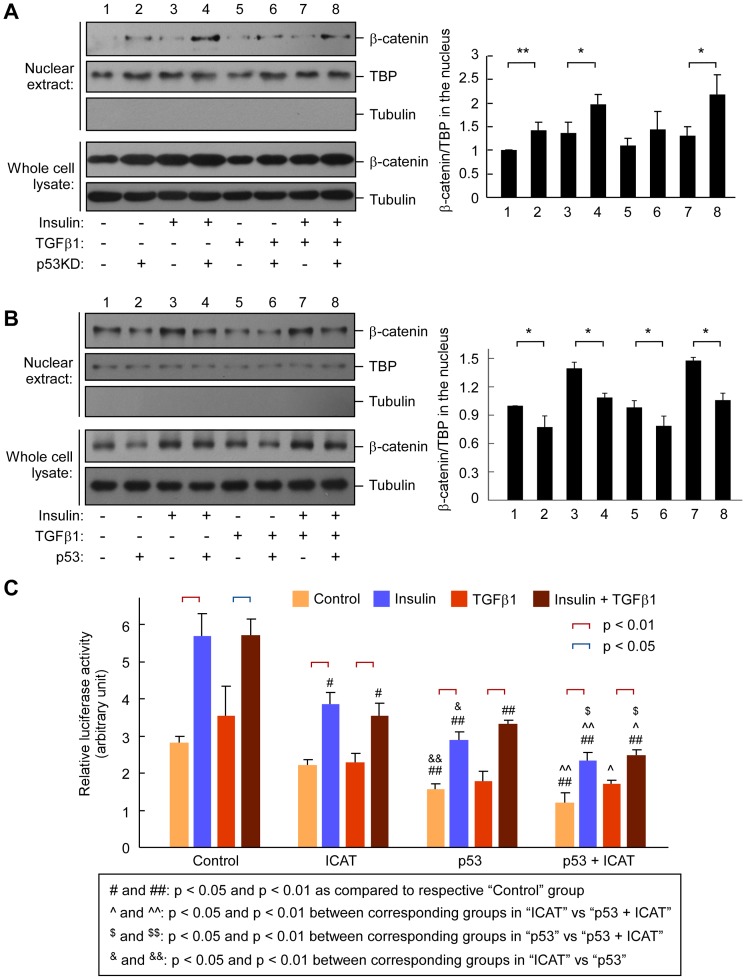
Alteration of nuclear accumulation and transcriptional activity of β-catenin by p53. (**A, B**) Effect p53 knockdown and overexpression on nuclear accumulation of β-catenin. PVTT-1 cells with either p53 knockdown (**A**) or p53 overexpression (**B**) were used in immunoblotting to detect total and nuclear β-catenin using antibodies as indicated. The cells were treated with or without insulin (10 μg/ml) and/or TGF-β1 (5 ng/ml) for 48 h before harvesting. TBP (TATA-binding protein) was used as a nuclear loading control. Band intensity of nuclear β-catenin relative to TBP from 3 independent experiments were calculated and shown on the right (mean ± SD). (**C**) Effect of insulin, TGF-β1 and p53 overexpression on β-catenin-mediated transcriptional response. Hep3B cells were transfected with LEF reporter and a renilla control plasmid, together with expression plasmids of p53 and ICAT as indicated. Twenty four hours after transfection, the cells were treated with or without insulin and/or TGF-β1 for 24 h, followed by dual luciferase assay. The data are shown from three independent experiments. * and ** indicate p<0.05 and p<0.01 respectively.

The p53 protein was previously reported to be associated with downregulation of β-catenin and its transcriptional activity [33], [34]. In addition, β-catenin has been characterized as an important molecule involved in EMT of tumor cells [14], [15]. We hypothesized that p53-mediated regulation on EMT was mediated, at least partially, by β-catenin. To address this hypothesis, we first analyzed the accumulation of β-catenin in the nuclei (Figure 2A). Treatment of PVTT-1 cells with insulin or insulin plus TGF-β1 was able to induce nuclear accumulation of β-catenin and such effect was enhanced by p53 knockdown (Figure 2A). It is also noteworthy that p53 knockdown alone appeared to increase nuclear accumulation of β-catenin. Next, we investigated whether overexpression of p53 also had an effect on nuclear accumulation of β-catenin. Consistently, we found that p53 overexpression could attenuate nuclear accumulation of β-catenin in the presence of insulin and/or TGF-β1 (Figure 2B).

We also investigated the transcriptional activity of β-catenin using a β-catenin-specific luciferase reporter [28]. We used Hep3B liver cancer cells instead of PVTT-1 cells as the later already contained a luciferase reporter [25]. Hep3B cells were p53 null [35]. We firstly confirmed that p53 and ICAT could be efficiently overexpressed in these cells (Figure S3 in File S1). We found that only insulin but not TGF-β1 treatment was able to stimulate the transcriptional activity of β-catenin (Figure 2C). As expected, expression of ICAT, a specific inhibitor of β-catenin pathway [36], could significantly reduce the basal and insulin-mediated transcriptional response. Overexpression of p53 could further reduce basal and insulin-stimulated transcriptional activity of β-catenin pathway. Furthermore, the transcriptional activity of β-catenin reached the minimum when ICAT and p53 were both overexpressed. Collectively, these data indicate that p53 and β-catenin have a functional crosstalk in liver cancer cells, *i.e*, p53 negatively regulates nuclear accumulation of β-catenin and β-catenin-mediated transcriptional response.

### Enhanced EMT by p53 knockdown is abrogated by inhibition of β-catenin signaling

**Figure 3:**
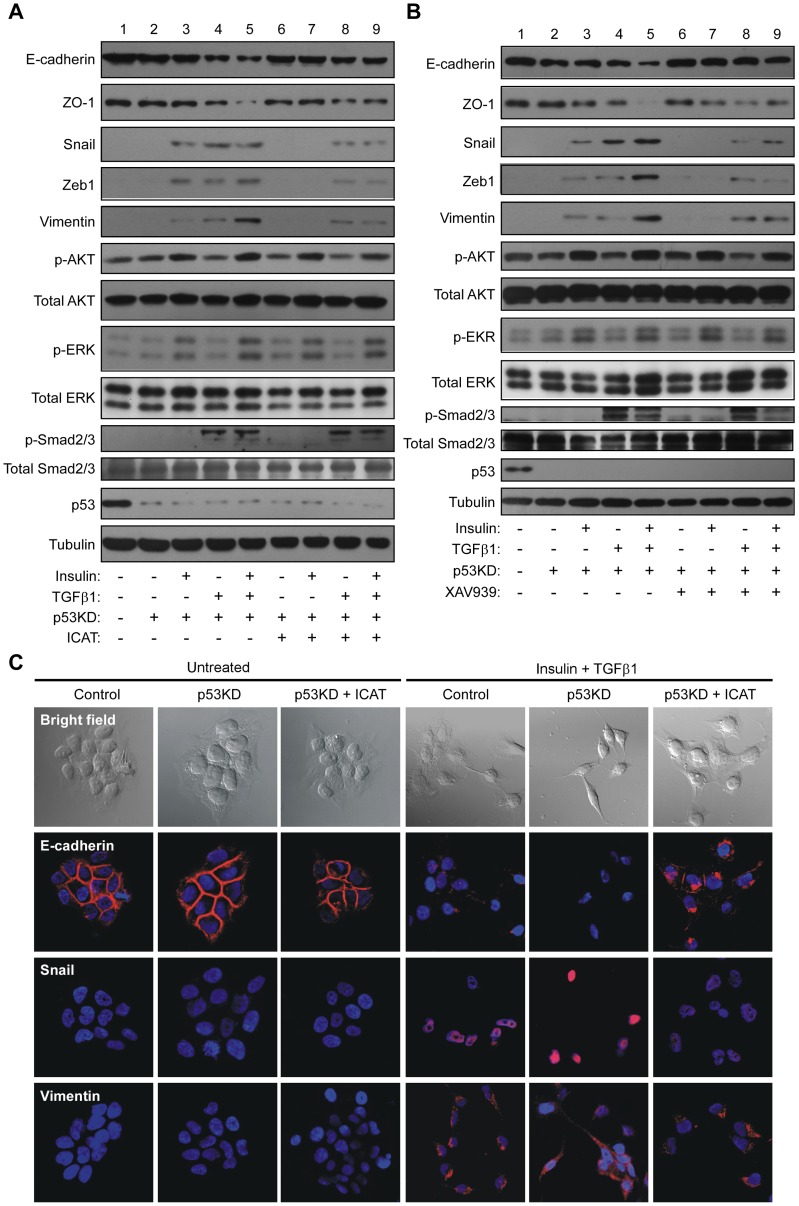
EMT enhanced by p53 knockdown is abrogated by inhibition of β-catenin pathway. Wild type or p53-silenced (p53KD) PVTT-1 cells were treated with or without insulin (10 μg/ml) and/or TGF-β1 (5 ng/ml) for 48 h before harvesting for immunoblotting using the antibodies as indicated. The cells were either overexpressed with ICAT **(A**) or treated with 1 µM of XAV939 for 48 h (**B**). The morphological changes of liver cancer cells were also analyzed (**C**). The cells were fixed and subjected to immunofluorescence staining to detect expression of E-cadherin, Snail and vimentin (red). The nuclei were stained with Hoechest 33342 (blue). The pictures of bright field were also shown.

As p53 is able to regulate the function of β-catenin, we next addressed an issue whether p53 regulation on EMT is mediated by β-catenin. PVTT-1 cells were treated with insulin and/or TGF-β1 in the presence or absence of p53 knockdown. Consistent with the results shown in Figure 1, p53 knockdown greatly sensitized insulin- and TGF-β1-induce EMT, shown as robust downregulation of E-cadherin and ZO-1 and upregulation of Snail, Zeb1 and vimentin (Figure 3A). Interestingly, the maximal EMT phenotype induced by p53 knockdown upon insulin/TGF-β1 treatment was partially abrogated by overexpression of ICAT. In ICAT-overexpression and p53 knockdown cells, the mRNA levels of p53 and ICAT were ∼32% and ∼210 folds of the control cells respectively by analysis with quantitative RT-PCR (Figure S4 in File S1). All the changes of E-cadherin, ZO-1, Snail, Zeb1, and vimentin induced by p53 knockdown and insulin/TGF-β1 treatment were partially diminished by ICAT overexpression (Figure 3A).

We also employed a specific inhibitor for β-catenin signaling to elucidate whether the p53 regulation on EMT is mediated by β-catenin. XAV939 has been found to antagonize *wnt* signaling through stimulating β-catenin degradation and stabilizing axin [37]. We found that the maximal changes of the EMT markers induced by p53 knockdown and insulin/TGF-β1 treatment could be abrogated by XAV939 (Figure 3B). Taken together, these results indicate that the EMT phenotype of PVTT-1 cells maximized by p53 knockdown and insulin/TGF-β1 treatment is, at least partially, mediated by β-catenin signaling pathway.

Epithelial cells undergoing EMT is featured by fibroblastoid-like morphology (4). To further confirm that the EMT induced by p53 knockdown is mediated by β-catenin pathway, we applied immunoﬂuorescence study to examine the morphological changes of PVTT-1 cells as well as the expression of a few EMT markers. As expected, we observed neither change in cell shape nor alteration in the expression of E-cadherin, Snail and vimentin upon p53 knockdown in the absence of insulin/TGF-β1 treatment (Figure 3C). However, treatment of the cells with insulin and TGF-β1 could induce a fibroblastoid-like morphology of the cells, together with reduction of E-cadherin expression and increase in Snail and vimentin expression. Furthermore, such changes of cell morphology and EMT markers appeared to be further enhanced by p53 knockdown. On the other hand, the changes of cell shape and EMT markers were almost completely abolished by ICAT overexpression. Therefore, these data further corroborated our observations that the EMT phenotype induced by p53 knockdown and insulin/TGF-β1 treatment is mediated by β-catenin signaling pathway.

### TGF-β1- and insulin-induced cell migration is enhanced by p53 knockdown and abrogated by inhibition of β-catenin signaling

**Figure 4:**
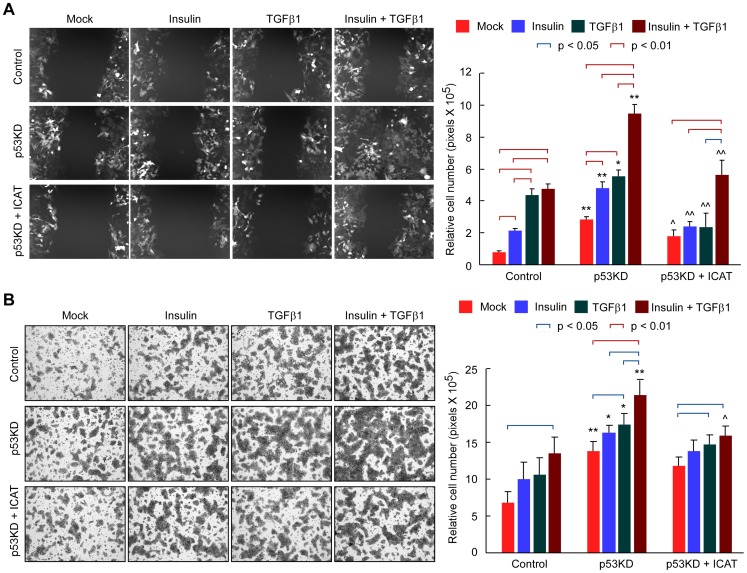
Liver cancer cell migration is enhanced by p53 knockdown and abrogated by inhibition of β-catenin pathway. PVTT-1 cells expressing GFP-tagged p53 shRNA (p53KD) and/or ICAT were treated with insulin (10 μg/ml) and TGF-β1 (5 ng/ml) as indicated and used in a wound healing assay (**A**) and a transwell assay (**B**). For wound healing assay, representative images of GFP-positive cells at 48 h after wound scratching are shown (X200) and the results from three independent experiments are shown in the right as mean ± SD. For transwell assay, the migrated cells on the bottom surface of each well were fixed and stained and the representative images are shown (X100). The area of migrated cells per field was quantified and shown in the right as mean ± SD (n = 8). * and ** indicate p<0.05 and p<0.01 respectively between the corresponding groups in “Control” *vs* “p53KD”. ? and ?? indicate p<0.05 and p<0.01 respectively between the corresponding groups in “p53KD” *vs* “p53KD + ICAT”.

Since we found that p53 knockdown could profoundly sensitize insulin/TGF-β1-induced EMT, we next investigated whether cell migration of liver cancer cells is also modulated by p53. In a wound healing assay, treatment of PVTT-1 cells with insulin or TGF-β1 could significantly enhance cell migration (Figure 4A). Furthermore, p53 knockdown significantly elevated the basal and insulin/TGF-β1-induced cell migration. However, the effect of p53 knockdown to promote cell migration was significantly abrogated by ICAT overexpression. We also analyzed cell migration using a transwell assay (Figure 4B). In general, we found that insulin/TGF-β1-stimulated cell migration was significantly enhanced by p53 knockdown and such effect was partially lessened by ICAT overexpression. Collectively, these data suggest that p53 can modulate the motility of liver cancer cells in combination with activation of PI3K/AKT and TGF-β pathways. Furthermore, the effect of p53 on cell migration is mediated by β-catenin pathway.

### Systemic metastasis is enhanced by p53 knockdown and abrogated by inhibition of β-catenin

**Figure 5:**
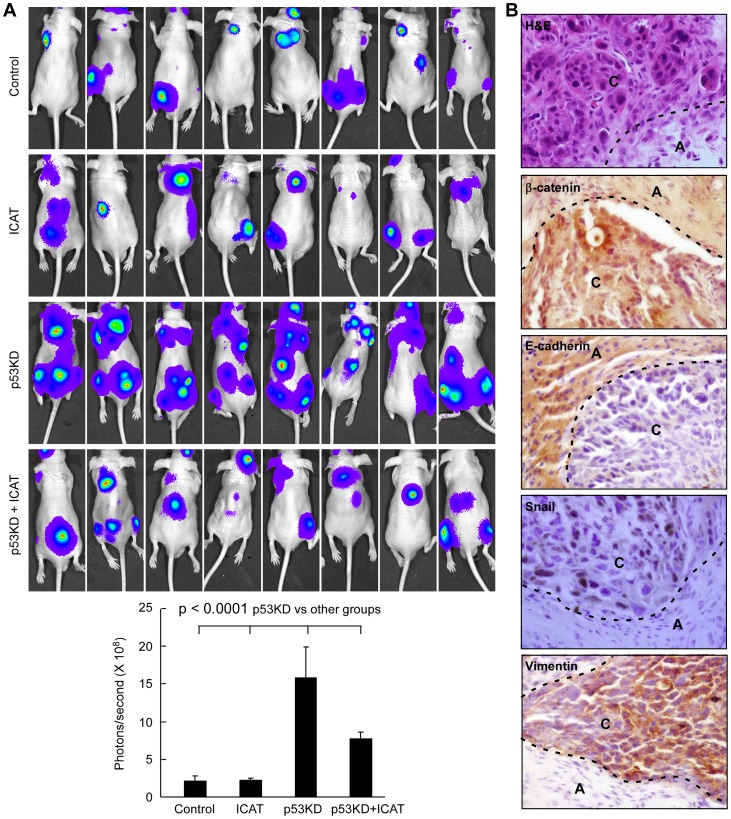
Systemic metastasis of liver cancer cells is enhanced by p53 knockdown and abrogated by inhibition of β-catenin pathway. PVTT-1 cells selected for the expression of ICAT and p53 shRNA (p53KD) were injected into the left ventricle of nude mice (2×10^5^ cells/mouse, n = 8 for each group). Luciferase expression was determined in 4 weeks after the injection (**A**). The statistic results are shown in the lower panel (mean ± SD). The metastatic foci in p53KD group were used in histological and immunohistochemistry analyses (**B**). The dotted line delimitates the border between metastatic cancer (marked as C) and adjacent normal tissue (marked as A). All the images are in 400X.

We next analyzed whether p53 had a direct effect on metastasis of PVTT-1 cells. We injected PVTT-1 cells infected with different expression lentivirus into the left ventricle of six-week-old nude mice and investigated tumor cell metastasis in 4 weeks. As previously reported, PVTT-1 is a human liver cancer cell line with relatively high metastatic potential [25], [31]. Equal number of cells was injected into each mouse as confirmed by *in vivo* imaging upon D-luciferin injection. As shown in Figure 5A, wild type PVTT-1 cells were able to metastasize to different areas of the body and ICAT overexpression had no effect on the “basal” metastasis of these cells. Intriguingly, p53 knockdown could robustly enhance metastasis of PVTT-1 cells, with a 7–8 fold increase in metastasis potential (Figure 5A). Furthermore, the promoting effect of p53 knockdown on liver cancer metastasis could be significantly abrogated by ICAT overexpression (Figure 5A). In summary, these data indicate that p53 has a profound effect on liver cancer metastasis and such effect is mediated by β-catenin signaling pathway.

To further analyze the properties of the metastatic cancers induced by p53 knockdown in these mice, we analyzed the cell morphology by hematoxylin-eosin (H&E) staining as well as the expression of β-catenin and EMT markers by immunohistochemistry (Figure 5B). The metastatic foci were featured by malignant liver cancer cells with heteromorphic nuclei. In comparison with adjacent normal tissue, the metastatic cells had nuclear accumulation of β-catenin, togsether with loss of E-cadherin and gain of Snail and vimentin. Collectively, these changes are consistent with our observations that p53 knockdown is able to induce EMT features in the liver cancer cells.

### Intrahepatic metastasis is enhanced by p53 knockdown and abrogated by inhibiting β-catenin pathway

**Figure 6:**
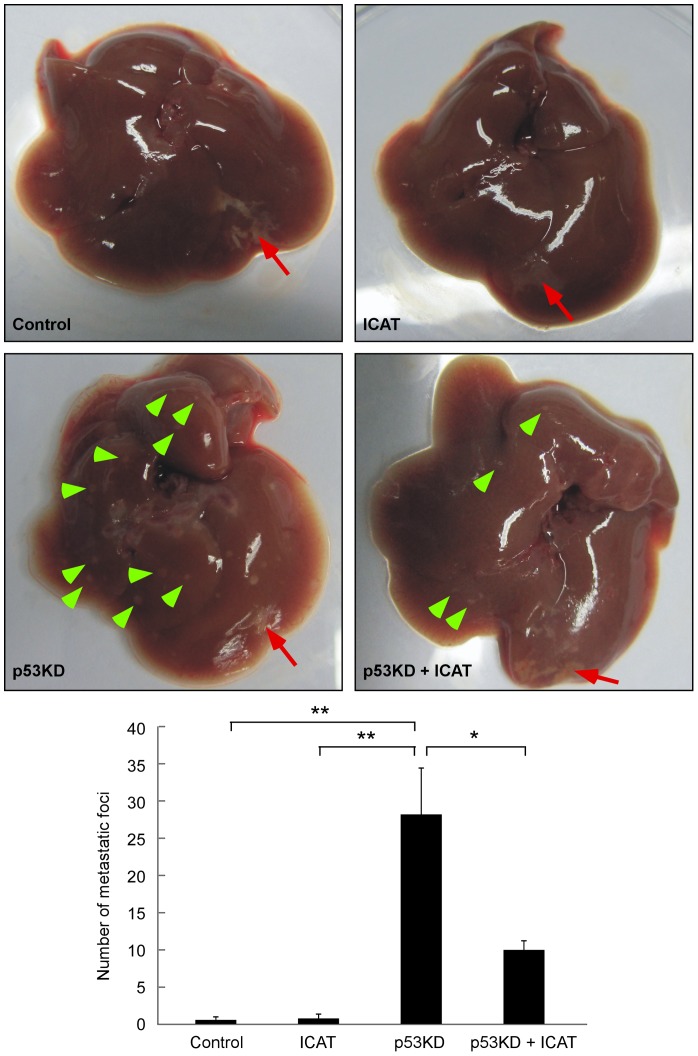
Intrahepatic metastasis of liver cancer cells is enhanced by p53 knockdown and reduced by inhibiting β-catenin pathway. PVTT-1 cells selected for the expression of p53 knockdown (p53KD) and ICAT overexpression were injected into the left hepatic lobe of nude mice (4×10^5^ cells/mouse, n = 5 for each group). Intrahepatic metastasis was determined at 6 weeks after injection by counting the metastatic foci in the uninjected liver lobes. Red arrow indicates the injection site and green arrowheads indicate metastatic foci. The statistic results are shown in the lower panel (mean ± SD) with * and ** indicating p<0.05 and p<0.01 respectively.

Finally, we employed an orthotopic mouse model to analyze whether p53 had an effect on intrahepatic metastasis. PVTT-1 cells infected with different lentivirus were injected into the left hepatic lobe in 5-week-old male nude mice and intrahepatic metastasis was determined in 6 weeks after the injection. As shown in Figure 6, we did not find obvious metastatic foci in the uninjected liver lobes in the mice injected with wild type or ICAT-overexpressing PVTT-1 cells. Intriguingly, p53 knockdown could robustly enhance intrahepatic metastasis of PVTT-1 cells, with an average of 28 metastatic foci per mouse (Figure 6). Furthermore, the enhancement of p53 knockdown on intrahepatic metastasis could be abrogated by ICAT overexpression (Figure 6). Under the condition without p53 knockdown, we did not observe visible metastasis in extrahepatic regions in the animals. However, we found putative extrahepatic metastasis in the mice injected with p53-downregulated PVTT-1 cells (Figure S5 in File S1). In summary, these data indicate that p53 has a profoundly negative effect on liver cancer metastasis and such effect is mediated by β-catenin signaling pathway.

## Discussion

In this study, we investigated the complex interplay among several important pathways implicated in EMT and metastasis of liver cancer cells. The involved signaling pathways are p53, PI3K/AKT signaling (initiated by insulin treatment), TGF-β signaling, and β-catenin pathway. In general, we found that both PI3K/AKT and TGF-β signaling pathways are able to slightly stimulate EMT and cell migration. However, the PI3K/AKT- and TGF-β-stimulated EMT and cell migration as well as liver cancer metastasis are robustly enhanced by p53 knockdown. Furthermore, β-catenin pathway is instrumental to the effect of p53 on EMT and metastasis of liver cancer cells. These studies are of great clinical significance as alterations of genes within these signaling pathways are commonly found in human HCC. The main mutations found in HCC include p53 gene (present in about 25–40% of cancers, depending on tumor stage), and β-catenin gene (about 25%, predominantly in HCV-related hepatocellular carcinoma) [1]. The gene of p53 is the most frequently mutated one in HCC, resulting in either loss of function or gain of new function [19]–[22]. In addition, alteration of genes in PI3K/AKT pathway is commonly found in HCC [2]. However, how these signaling pathways interact with each other has not been fully elucidated. Furthermore, although some of these signaling pathways such as TGF-β have been demonstrated to play a role in EMT, currently it is unknown how these pathways are coordinated to promote EMT and metastasis. Our study, therefore, has provided a piece of critical evidence about the interplay between these pathways in EMT and tumor metastasis.

In this work, we reveal that p53 plays a vital role in EMT and metastasis of liver cancer cells. Firstly, insulin- and TGF-β1-induced changes of critical EMT markers such as E-cadherin, ZO-1, Snail, Zeb1, and vimentin were greatly sensitized by p53 knockdown in liver cancer cells. Secondly, insulin- and TGF-β1-stimulated migration of liver cancer cells was significantly enhanced by p53 knockdown. Finally, *in vivo* metastasis of liver cancer cells were robustly enhanced by p53 knockout. Therefore, these findings demonstrate that p53 has a critical EMT checkpoint function, in addition to its well characterized functions in the regulation of cell cycle, apoptosis and genomic stability [23]. It was found that loss or inhibition of p53 function prevents cellular apoptosis and contributes to HCC development [24]. However, whether p53 plays a functional role in EMT and metastasis of liver cancer has not been fully elucidated. Interestingly, our results are consistent with our recent report showing that p53 plays an active role in EMT in the formation of spontaneous skin cancer-like tumors in the mice [26]. In addition, our study is consistent with the findings that the absence of p53 gene does not increase tumor incidence in an inducible HCC mice model, while elevating the ability of the tumors to invade and metastasize to lungs and bile ducts [38], [39]. Furthermore, a few recent studies have uncovered the possible relationship between p53 and EMT. Loss of p53 could cooperate with activated Ras to promote cell motility through activating RhoA [40]. Pancreatic acinar cells from p53^−/−^ mice can undergo EMT upon subculture [41]. In mammary epithelial cells and HCC cell lines, p53 regulates EMT by up-regulating the expression of miR-200, thereby repressing Zeb1 and Zeb2 [42], [43]. In particular, our work is consistent with the findings that p53 upregulates miR-200 which in turns downregulates Zeb1 and Zeb2 and reduces EMT in liver cancer cells [43]. Loss of p53 in colon, breast, and lung carcinoma cells activates Snail1-dependent EMT through decreasing miRNA-34 levels [44]. In our study, we did find a decreased expression of miR-200 family members when p53 was silenced (Figure S6 in File S1), further supporting the idea that p53 may influence EMT through regulating miR-200 family. Recently it was reported that Twist opposes p53 function in human prostate cancer cells and breast cancer cells through direct interaction with p53, indicated that p53 may have an inhibitory effect on EMT [45]. In addition, wild type p53 upregulates MDM2 and forms a p53-MDM2-Slug complex to degrade Slug in non-small-cell lung cancer cells, and Slug expression level correlates positively with lung adenocarcinoma metastasis in HCC patients [46]. As more studies are forthcoming in this direction, we predict that p53 will soon be recognized as one of the most pivotal factors in dictating EMT and tumor metastasis.

Another important finding in this study is that p53 regulation on EMT and metastasis of liver cancer cells are, at least partially, mediated by β-catenin signaling pathway. The p53 was able to modulate nuclear accumulation and transcriptional activity of β-catenin in PVTT-1 cells. The changes of EMT markers upon p53 knockdown were abrogated by inhibition of β-catenin signaling pathway using either ICAT overexpression or administration of a chemical β-catenin inhibitor. The cell migration enhanced by p53 knockdown was inhibited by ICAT overexpression. Furthermore, metastasis of liver cancer cells by p53 knockdown was significantly lessened by ICAT overexpression. Interestingly, activating mutations of β-catenin mutations and aberrant accumulation of β-catenin are frequently observed in human HCC. It has been shown that wild-type β-catenin accumulation in HCC cells is associated with mutational inactivation of p53 gene [47]. It was also observed that p53 and β-catenin mutation rates are inversely correlated in HCC, suggesting that inactivation of p53 is an important cause of aberrant accumulation of β-catenin in cancer cells [47]. Consistently, our study also pinpoints β-catenin as a critical molecule implicated in p53-mediated regulation on EMT and metastasis of liver cancer cells. It is noteworthy that β-catenin pathway alone is not sufficient to sustain HCC development. Liver specific expression of activated form of β-catenin in transgenic mice only displays hepatomegaly but not HCC [48], suggesting that other factor(s) is needed to cooperate with β-catenin activation for HCC development. Therefore, it is important to explore the molecular mechanism underlying the cross-talk between p53 and β-catenin in the future. As p53 is frequently mutated or lost in human HCC and p53 is actively involved in metastasis via regulation on β-catenin signaling as shown in this study, we postulate that inhibition of β-catenin pathway would be an effective therapeutic strategy to control metastasis of human HCC.

## Supporting Information

File S1 (PDF)
