# Supplementary material for: Critical Roles of p53 in Epithelial-Mesenchymal Transition and Metastasis of Hepatocellular Carcinoma Cells
Source: PLoS One. 2013 Sep 2;8(9):e72846. doi: 10.1371/journal.pone.0072846 (PMC3759437; doi:10.1371/journal.pone.0072846)

Supporting Information

The Supplemental Materials contain 6 Supporting Figures

Figure S1. Efficiency of p53 knockdown for Figure 1A

PVTT-1 cells infected with or without p53 knockdown lentivirus were used in real-time RT-PCR to analyze the expression level of p53. The data are as mean ± SD.

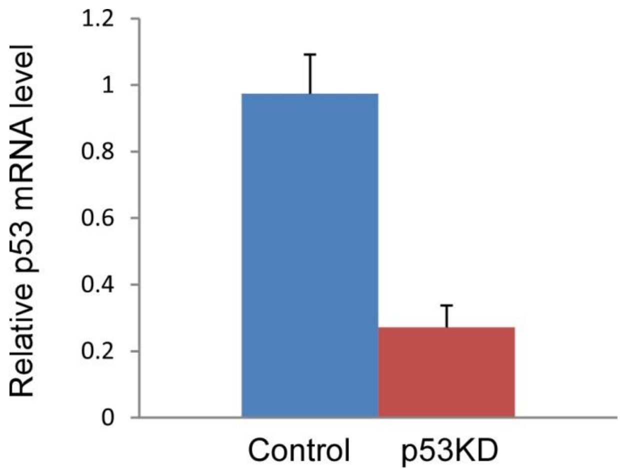

Figure S2. TGF-β1- and insulin-induced EMT is enhanced by p53 knockdown in HepG2 cells

HepG2 cells infected with lentivirus expressing p53 shRNA were treated with or without insulin (10 μg/ml) and/or TGF-β1 (5 ng/ml) for 48 h. The cell lysate was used in immunoblotting with the antibodies as indicated.

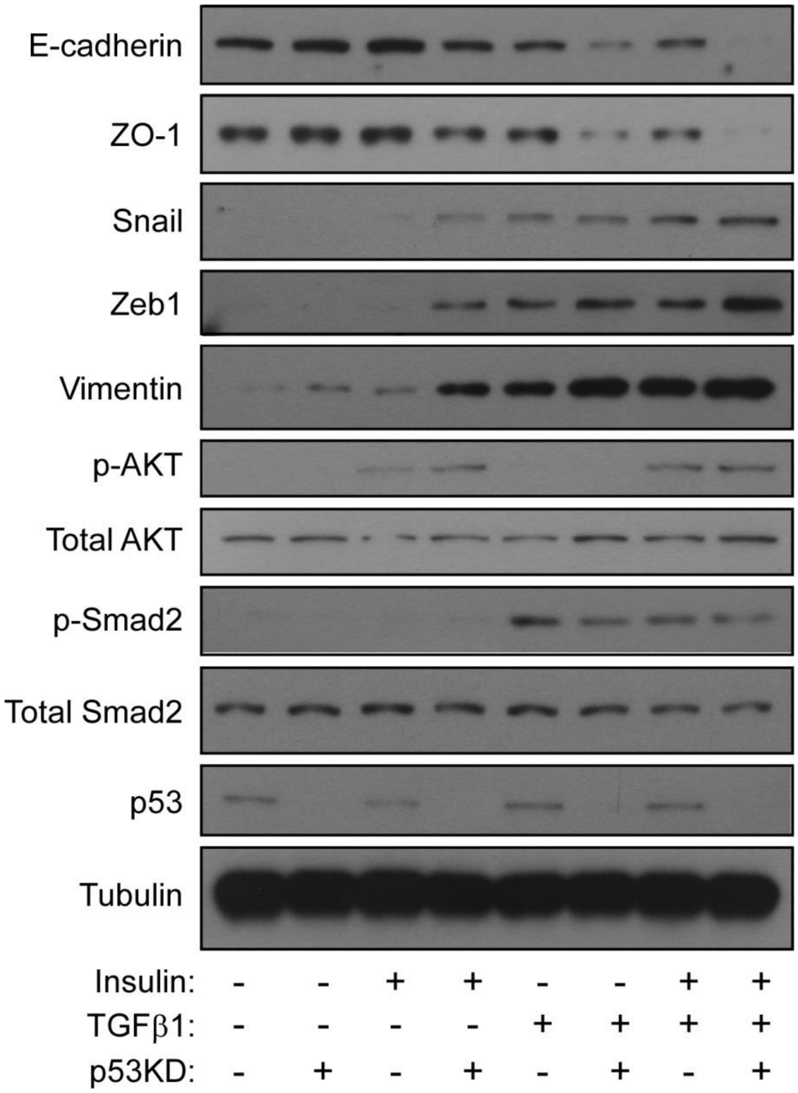

Figure S3. Confirmation of p53 and ICAT overexpression in Hep3B cells (for Figure 2C)

Hep3B cells were transiently transfected with plasmids expressing p53 and/or ICAT as indicated. At 48 hours after transfection, the cell lysate was used in Western blot to analyze the expression of p53 (A). Real-time RT-PCR was used to analyze the mRNA level of ICAT (B).

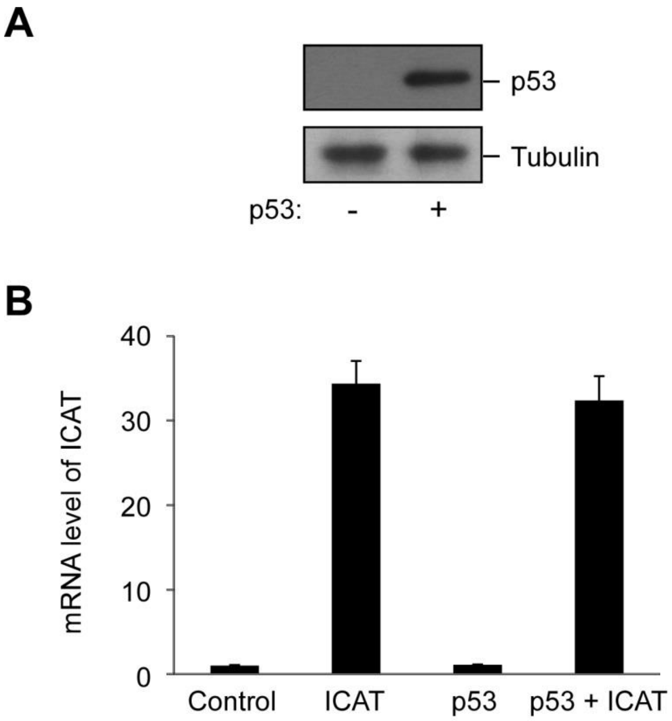

Figure S4. Efficiency of p53 knockdown for Figure 3A

PVTT-1 cells transfected with or without p53 knockdown plasmid were used in real-time RT-PCR to analyze the expression level of p53. The data are as mean ± SD.

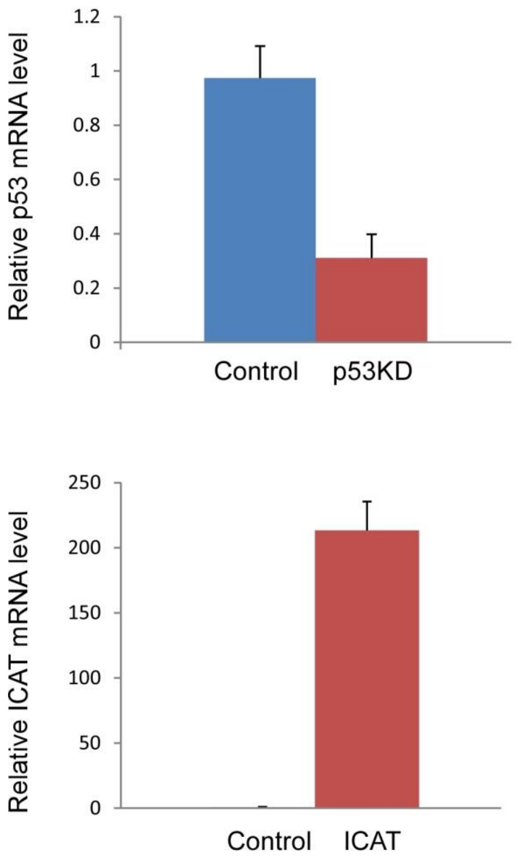

Figure S5. ***In vivo*** **imaging of orthotopic mice model.**

PVTT-1 cells selected for the expression of ICAT and p53 shRNA (p53KD) were injected into the left hepatic lobe of nude mice (4 x 10^5 cells/mouse, n = 5 for each group). Luciferase expression was determined at 6 weeks after the injection. Red arrows indicate putative extrahepatic metastatic lesion.

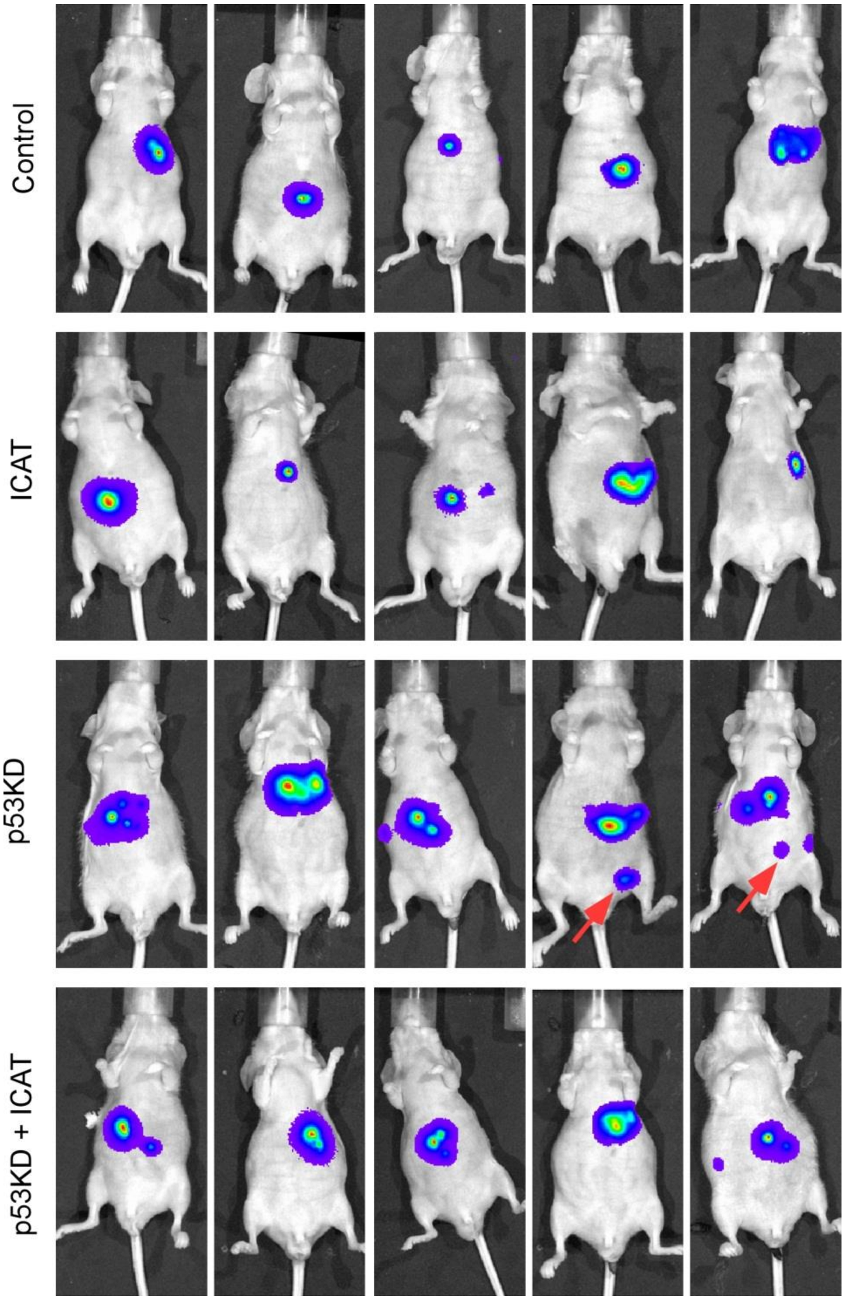

Figure S6. Knockdown of p53 downregulates the expression of miR-200 family members

PVTT-1 cells with or without p53 knockdown were treated with or without insulin (10 μg/ml) and TGF-β1 (5 ng/ml) for 24 h. The cells were then harvested for microRNA analysis by real-time PCR. The data are calculated from triplicate experiments and shown as mean ± SD. * and ** indicate $p < 0.05$ and $p < 0.01$ respectively.

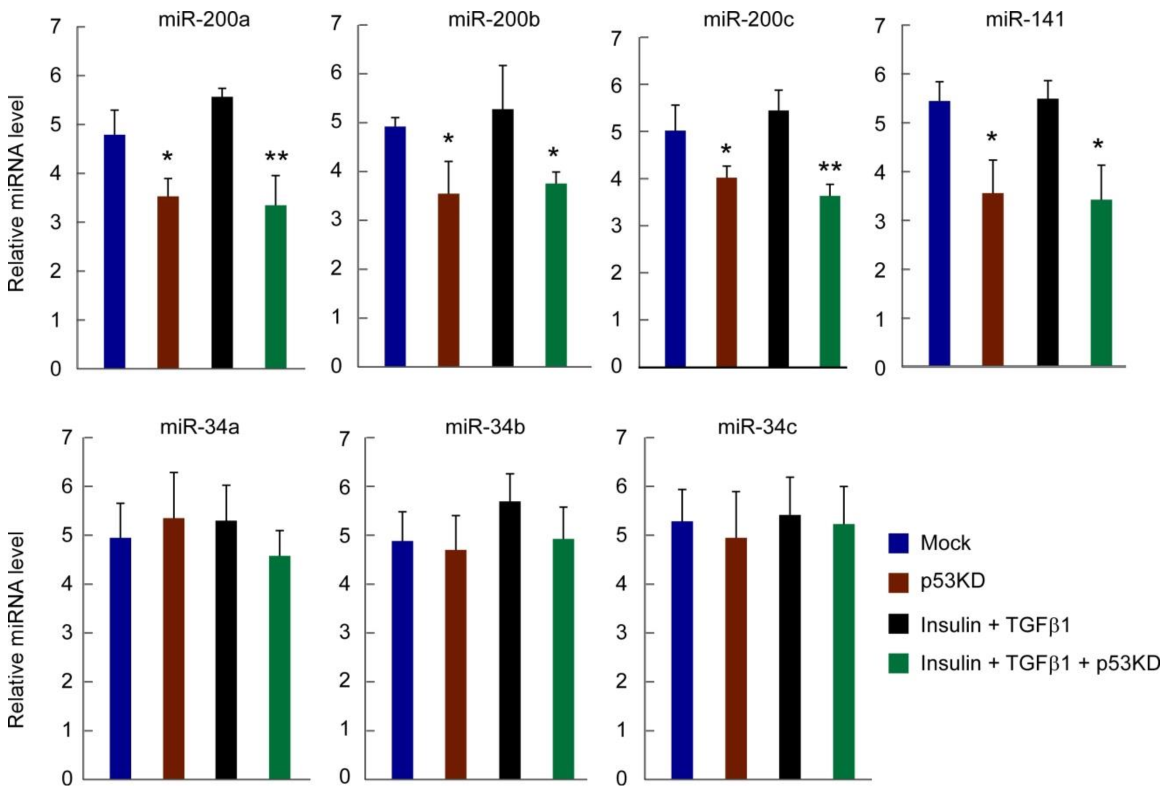

Supplement: File S1 — (PDF) [file pone.0072846.s001.pdf]
